# Patient-Derived Xenograft and Organoid Models for Precision Medicine Targeting of the Tumour Microenvironment in Head and Neck Cancer

**DOI:** 10.3390/cancers12123743

**Published:** 2020-12-12

**Authors:** Tet Woo Lee, Amy Lai, Julia K. Harms, Dean C. Singleton, Benjamin D. Dickson, Andrew M. J. Macann, Michael P. Hay, Stephen M. F. Jamieson

**Affiliations:** 1 Auckland Cancer Society Research Centre, University of Auckland, Auckland 1023, New Zealand; tw.lee@auckland.ac.nz (T.W.L.); amy.lai@auckland.ac.nz (A.L.); j.harms@auckland.ac.nz (J.K.H.); d.singleton@auckland.ac.nz (D.C.S.); b.dickson@auckland.ac.nz (B.D.D.); m.hay@auckland.ac.nz (M.P.H.); 2 Maurice Wilkins Centre for Molecular Biodiscovery, University of Auckland, Auckland 1010, New Zealand; amacann@adhb.govt.nz; 3 Department of Pharmacology and Clinical Pharmacology, University of Auckland, Auckland 1023, New Zealand; 4 Department of Radiation Oncology, Auckland City Hospital, Auckland 1023, New Zealand

**Keywords:** head and neck squamous cell carcinoma, precision medicine, tumour microenvironment, hypoxia, patient derived xenografts, organoids

## Abstract

**Simple Summary:**

The identification and validation of strategies to individualise therapy of patients with head and neck squamous cell carcinoma require cellular and animal models that accurately represent the complex microenvironment in which human tumours grow. Recently, patient-derived xenograft and organoid models of head and neck squamous cell carcinoma have been established that recapitulate the morphology, genetics and response to therapy of the human tumours they originated from. In this review, we discuss the development of patient-derived xenograft and organoid models of head and neck squamous cell carcinoma and describe their ability to predict clinical response to therapy. We focus on the utility of these tools to enable greater precision of both approved and experimental medicines through individualised therapeutic approaches.

**Abstract:**

Patient survival from head and neck squamous cell carcinoma (HNSCC), the seventh most common cause of cancer, has not markedly improved in recent years despite the approval of targeted therapies and immunotherapy agents. Precision medicine approaches that seek to individualise therapy through the use of predictive biomarkers and stratification strategies offer opportunities to improve therapeutic success in HNSCC. To enable precision medicine of HNSCC, an understanding of the microenvironment that influences tumour growth and response to therapy is required alongside research tools that recapitulate the features of human tumours. In this review, we highlight the importance of the tumour microenvironment in HNSCC, with a focus on tumour hypoxia, and discuss the fidelity of patient-derived xenograft and organoids for modelling human HNSCC and response to therapy. We describe the benefits of patient-derived models over alternative preclinical models and their limitations in clinical relevance and how these impact their utility in precision medicine in HNSCC for the discovery of new therapeutic agents, as well as predictive biomarkers to identify patients’ most likely to respond to therapy.

## 1. Head and Neck Squamous Cell Carcinoma

Head and neck squamous cell carcinoma (HNSCC) describes a subset of cancers, which begin in the squamous epithelial cells lining the mucosal surfaces of the head and neck region, including the oral and nasal cavities, pharynx, and larynx. HNSCC accounts for over 90% of all head and neck cancers [1], which collectively are the seventh most common cancer presentations and cause of cancer-related mortality worldwide with an approximate 900,000 new cases and 450,000 deaths in 2018 [2]. The major risk factors for HNSCC are frequent use of alcohol and tobacco, genetic susceptibilities, and infection with certain strains of human papillomavirus (HPV) [3,4,5,6]. Meta-analyses have revealed approximately 20–30% of HNSCC cases are HPV-positive, with the greatest prevalence of HPV-positivity in the oropharynx [7,8].

HNSCC carcinogenesis is driven by frequent chromosomal instability and gene mutations that differ with HPV infection status [9]. The primary somatic genomic alterations in HPV-negative disease include mutations in *TP53*, *PIK3CA*, *FAT1*, and *NOTCH1*, loss of *CDKN2A*, and amplification of *CCND1* [9,10], implicating loss of cell cycle control and enhanced WNT signalling as major drivers of disease [11]. A subgroup of predominantly oral cavity HPV-negative tumours have a low frequency of copy number alterations, are wild-type for *TP53*, and likely follow an alternative tumourigenesis pathway driven by activating *HRAS* and inactivating *CASP8* mutations as a result of ageing rather than high exposure to tobacco and alcohol [9,12]. Common genetic alterations in HPV-positive HNSCC include loss of *TRAF3*, amplification of *E2F1*, and a higher rate of *PIK3CA* mutations than HPV-negative tumours. The overall mutational burden is lower than HPV-negative disease [13], with few alterations in *TP53* and *CDKN2A* [9,10], since its aetiology is driven by HPV infection, predominantly by HPV-16 and to a lesser extent HPV-18. HPV infection integrates viral oncogenes E6 and E7 into host cells and induces degradation or inactivation of tumour suppressor proteins p53 and Rb [14,15], essentially phenocopying the *TP53* and *CDKN2A* genetic events seen with HPV-negative disease.

Locally advanced HNSCC is treated with surgery or radiotherapy with curative intent. Frequently these modalities are supplemented with concurrent platinum-based chemotherapy [16] or with the EGFR inhibitor cetuximab [17] as many tumours overexpress EGFR. [18]. Metastatic or unresectable recurrent disease is treated first-line with cetuximab and chemotherapy [19] or pembrolizumab, either as a single agent in patients with tumours expressing PD-L1, or in combination with platinum chemotherapeutics and 5-fluorouracil regardless of PD-L1 status [20]. Pembrolizumab and another immune checkpoint inhibitor, nivolumab, are also used for second line therapy of recurrent/metastatic HNSCC following disease progression on platinum-based chemotherapy [21,22]. HPV-positive disease is typically more responsive to treatment and thus has a more favourable prognosis than HPV-negative disease [21,23,24]. Despite the recent approvals of these immune checkpoint inhibitors in HNSCC, objective response rates remain low at second-line [21,22], and similar to cetuximab and chemotherapy at first-line [20]. Ultimately, new therapies are required to improve outcomes in patients unresponsive to current treatments.

## 2. Precision Medicine in HNSCC

Precision medicine to tailor treatment plans to individual patients based on expression of predictive biomarkers is becoming more widespread in oncology as the use of diagnostics identifying treatment-sensitive patient subpopulations rises. The recent definition of a biomarker as a “defined characteristic that is measured as an indicator of normal biological processes, pathogenic processes or responses to an exposure or intervention, including therapeutic interventions” [25] does little to aid the researcher in the quest for tools to guide patient selection for treatment or response to that treatment. These biomarkers can be molecular, histologic, radiologic, or physiologic in nature. In the case of HNSCC, as for other tumour types, an ideal biomarker would not only describe the molecular aberrations within the tumour cells that drive carcinogenesis and present targets for therapeutic intervention, but also reflect the characteristics of the tumour microenvironment that have an impact on disease progression and treatment response. However, the implementation of precision medicine approaches in HNSCC remain limited. Cetuximab is widely used to treat HNSCC, yet therapy is not individualised based on EGFR expression, unlike for EGFR inhibitors in other cancers, since EGFR copy number does not act as a predictive biomarker for the efficacy of cetuximab in HNSCC [26]. Similarly, second-line nivolumab and pembrolizumab and first-line pembrolizumab in combination with chemotherapy are also approved in all patients, despite the targeted nature of these therapies. The only therapy for HNSCC that requires a diagnostic is single agent pembrolizumab at first-line, based on evidence of efficacy in patients with a PD-L1 combined positive score (CPS) ≥ 1 [20]. This recent biomarker-driven approval will likely herald a new era in HNSCC treatment, where new therapies diversify away from an all-comers strategy to a precision medicine approach where only the subpopulation of patients most likely to benefit will receive treatment of targeted therapies. Critical to the use of these biomarkers is the co-development of a diagnostic in conjunction with the therapeutic agent.

### 2.1. The Importance of Tumour Hypoxia as a Prognostic Factor in HNSCC

Key to enabling precision medicine in HNSCC is an understanding of the microenvironment that influences HNSCC tumour growth and response to therapy. One of the most important negative prognostic markers in HNSCC is the presence of low oxygen levels, or hypoxia. Strong evidence of the importance of hypoxia in HNSCC initially came from studies using Eppendorf oxygen electrodes to directly measure oxygen levels (pO2) in late-stage primary and nodal head and neck tumours, with patients with hypoxic tumours tending to have worse overall survival and tumour control following radiotherapy or chemoradiotherapy [27,28,29,30]. These studies also showed the extent to which hypoxia is a factor in HNSCC, with 43–70% of tumours having a pO_2_ of <10 mmHg [27,28,29] and the median fraction of hypoxic (<5 mmHg pO_2_) regions among the tumours being 29–50% [27,28,30]. More recently, hypoxia gene signatures have been used to show that HNSCC tumours are the most hypoxic out of 19 common tumour types [31].

There are a number of mechanisms that underlie the poor prognosis of hypoxic tumours. One of the most-well known, and first to receive widespread attention, is the resistance of hypoxic cells to radiotherapy. Since radiation-induced DNA damage is an oxygen-dependent process, radiation doses required to kill hypoxic cells are two- to three-fold higher than those required to kill well-oxygenated cells [32]. Knowledge of the importance of oxygen to radiotherapy has provided an example of the use of a tumour microenvironment biomarker to individualise therapy—stratification of HNSCC patients according to a hypoxic gene expression classifier (Toustrup 15-gene signature) has shown that those with more hypoxic tumours derive greater benefit from the hypoxia-selective radiosensitiser nimorazole than those with less hypoxic tumours [33]. In addition to direct effects on radioresistance, hypoxia also contributes to poor prognosis by driving cellular expression programmes that lead to tumour progression. Hypoxia-inducible transcription factors HIF-1/2 regulate a transcriptional programme that results in extensive changes in the expression levels of numerous coding and non-coding RNA species resulting in phenotypic adaptation [34]. This transcriptional response drives disease progression and negative prognostic outcomes by promoting angiogenesis, genomic instability, and metastasis.

Recent research also indicates that tumour hypoxia enables immune evasion by promoting and sustaining an immunosuppressive tumour microenvironment. The hypoxic niche recruits various immune cells to the tumour including macrophages, neutrophils, myeloid-derived suppressor cells, and regulatory T cells [35,36,37]. Hypoxia influences the phenotype and activity of these immune cell populations by promoting tumour adenosine production by upregulation of adenosine-generating enzymes [38], and promotes downregulation of the MHC class I antigen processing pathway in a HIF-dependent manner [39]. A combined hypoxia and immune gene signature has been found to be highly prognostic in HNSCC datasets. Patients with hypoxia^low^/immune^high^ score had a favourable 5-year survival probability (71%) and were more likely to display an “inflamed” microenvironmental profile. In contrast, those defined as hypoxia^high^/immune^low^ had unfavourable 5-year survival probability (51%) and were more likely to display an “immune-desert” phenotype. Importantly, immunohistochemical staining confirmed an inverse correlation between these two features: tumours with greater CA9 staining (a marker of hypoxic tumour cells) had lower prevalence of intratumoural T cells and vice versa [40]. Clinical reports appear to confirm these observations with recurrent/metastatic HNSCC patients that have low levels of tumour hypoxia (CA9 % and staining intensity) demonstrating significantly greater response rates and overall survival when treated with anti-PD-1 therapies [41]. These results highlight how multiple aspects of the tumour microenvironment can influence patient response and that a detailed understanding of the contribution of the tumour microenvironment to tumour biology and treatment is therefore essential for the development and tailoring of treatments to HNSCC patients.

### 2.2. Targeting the Hypoxic Tumour Microenvironment in HNSCC

A range of strategies to modify hypoxia have been explored to counteract the negative consequences of tumour hypoxia to the prognosis of HNSCC treated with radiotherapy. Numerous clinical trials using nitroimidazole oxygen-mimetic radiosensitisers [42,43] or supplemental oxygen as hyperbaric oxygen or carbogen breathing combined with nicotinamide as a blood-flow modifier and accelerated radiotherapy (ARCON) [44,45,46] have been conducted since the 1960s. Although many of these trials failed to reach a conclusive result, a meta-analysis of these hypoxia modifications indicates an overall improvement in tumour control and patient survival [47]. The radiosensitiser nimorazole is currently being evaluated in a phase III trial in combination with accelerated chemoradiotherapy in HNSCC patients, prospectively exploring the value of the Toustrup 15-gene hypoxic signature [33] as a biomarker (NCT01880359).

A change in approach evolved in the 1980s; rather than try to modify hypoxia within solid tumours, hypoxia-activated prodrugs (HAPs) were developed to exploit the presence of tumour hypoxia as a selective drug delivery address. HAPs feature a chemical moiety that deactivates the drug until the prodrug is selectively metabolised in low-oxygen environments by one electron reductases in an oxygen-sensitive manner [48,49]. Early efforts focused on prodrugs of cytotoxic radical species such as mitomycin C [50] and tirapazamine [51]. Hypoxia can be detected non-invasively in patients by positron emission tomography (PET) imaging with ^18^F-labelled nitroimidazole probes [52,53]. A randomised phase II trial of chemoradiotherapy plus or minus tirapazamine in HNSCC confirmed the benefit of adding a hypoxia-targeting agent to chemoradiotherapy, but only for those patients with a positive ^18^F-misonidazole PET imaging biomarker signal [54].

Despite this clear indication of the value of biomarker-guided hypoxia targeting, subsequent clinical trials of HAPs have not used hypoxic biomarkers to stratify patients. For example, the ensuing phase III trial of tirapazamine with chemoradiation did not select HNSCC patients based on hypoxic status and failed to demonstrate any superiority over chemoradiation alone [55]. Analysis of a subgroup of patients within this trial imaged with a PET hypoxia biomarker (^18^F-fluoroazomycin arabinoside, FAZA) further demonstrated the prognostic potential of hypoxia biomarkers, with hypoxia-positive patients treated with chemoradiotherapy faring significantly worse than hypoxia-positive patients receiving tirapazamine plus radiotherapy [56]. The case for biomarkers was then reiterated in a retrospective analysis showing improved locoregional control and failure-free or overall survival in treatment-compliant patients [57] and patients with HPV p16-negative hypoxic tumours [58]. The mitomycin C HAP porfiromycin [50] and the irreversible EGFR inhibitor HAP tarloxotinib have also failed to show benefit in HNSCC clinical trials, although the failure of tarloxotinib may reflect not only a lack of biomarker support but the challenges of assessing HAPs as monotherapy when they are only targeting a subset of the tumour microenvironment. Likewise, evofosfamide failed in phase III trials in pancreatic cancer and soft tissue sarcoma [59], with its activity, previously observed in complementary phase II trials [60,61], undermined by the absence of patient stratification based on hypoxia biomarkers. Evofosfamide has, however, shown promising activity in HNSCC in preclinical models and a handful of patients [62,63], and an ability to enhance the efficacy of immunotherapies [62,64] by eliminating the immunosuppressive effects of hypoxia and promoting T cell proliferation and infiltration into tumours [64]. On that basis, a dose-escalation phase I trial of evofosfamide and ipilimumab was initiated that included one HNSCC patient who subsequently demonstrated a partial response [65].

Overall, while there is considerable evidence to justify targeting hypoxia in HNSCC, the clinical trials that have been performed to date suggest that it is imperative that future therapeutic strategies utilise prospective hypoxia biomarkers to guide their clinical development. As discussed above, the development of such therapeutic strategies also require a thorough understanding of the contribution of the different aspects of the tumour microenvironment (e.g., immune infiltration, gene mutation status) on treatment response, which, in turn, requires research tools that recapitulate these features of human tumours.

## 3. Patient-Derived Models of HNSCC

To enable translation of precision medicine approaches for treating HNSCC into the clinic, appropriate preclinical tumour models must be employed that mirror the complex microenvironment of human tumours. Tumour xenograft models derived from human cancer cell lines are widely used to model HNSCC, with well over 300 HNSCC cell lines described in the literature, although the vast majority of these are HPV-negative [66]. Many of these cell lines are early passage and can adequately represent common gene mutations observed in clinical HNSCC tumours [67,68,69]. However, cell lines typically undergo extensive adaptation under supraphysiological oxygen and nutrient culturing conditions and, as xenografts, frequently lose the structural, genetic, and phenotypic heterogeneity of the human tumour microenvironment and poorly model clinical response [70,71,72]. In recent years, patient-derived models of HNSCC, including patient-derived xenograft (PDX) and organoid models, have become more widespread in preclinical studies to model human disease.

### 3.1. Patient-Derived Xenograft Models of HNSCC

PDX models of HNSCC are generated through the direct implantation of intact tissue from human tumours into immunodeficient mice either subcutaneously or orthotopically into anatomically similar sites such as the floor of the mouth or the base of the tongue [73]. Initial HNSCC PDX models typically utilised athymic nude mice as hosts [74,75,76], but higher engraftment efficiencies have been observed with more highly immunodeficient mouse strains, such as NOD scid gamma (NSG) [77]. HNSCC PDX models have been generated by a number of academic research groups that report a range of engraftment efficiencies, in most cases around 60% (Table 1). This is exemplified by the largest reported HNSCC PDX biobank of 161 HPV-negative models at the Princess Margaret Cancer Centre in Toronto with an engraftment efficiency of 66% [78]. Engraftment rate is frequently higher for PDXs from patients with advanced lymph node metastases [75,78,79] and perineural invasion [80], but is independent of patient age, sex, or tumour stage [76,77,81]. Tumours that rapidly engrafted, within eight weeks, correlated with worse patient outcomes [78]. In addition to academic efforts, collections of HNSCC PDX tumour models have also been generated by large PDX consortia (e.g., EuroPDX, The Jackson Laboratory and the National Cancer Institute’s PDXNet, and Patient-Derived Models Repository) and contract research organisations (e.g., Champions Oncology, Crown Bioscience).

At the histopathological level, PDX tumours accurately recapitulate the heterogeneous and complex tumour microenvironment of HNSCC with retention of cell morphology, stromal and vascular architecture, abundant regions of keratinous cells, and matched degrees of differentiation to the parent tumour [63,75,76,78,79,81,86]. Although tissue histopathology closely resembles clinical tissue at early PDX passages, they tend to lose stroma and become more homogeneous in subsequent generations [75], which can alter tumour growth rates [94], although differentiation status remains stable [81]. Multiple studies have genetically characterised HNSCC PDX tumours and compared gene expression, gene mutation status, and DNA copy number to the donor clinical specimen or HNSCC population data from The Cancer Genome Atlas [9]. All studies report a high concordance in expression levels, mutation frequency, and copy number profiles between PDX models and HNSCC tumours across the whole genome or for particular oncogenic variants [75,76,78,79,82,83,84,85,88,92,93,95]. Although discordances in some genes were observed, gene expression patterns were more similar to patient tumours for PDX tumours than for HNSCC cell lines [75]. Similarly, the majority of proteins are conserved in PDX tumours, although some differences from clinical tumours arise through replacement of human stromal compartments and preferential selection of proteins associated with proliferative signalling [77,85].

The ability of PDX tumours to model patient tumour hypoxia is less established. Hypoxia is primarily determined by the tumour microvasculature [96], so in PDX models hypoxia is likely controlled more by the mouse host than the human tumour specimen. Therefore, PDX models are unlikely to recapitulate hypoxia of the donor patient tumour in all cases. We have reported that our own HNSCC PDX models have hypoxic fractions that are within the range of published clinical data based on immunostaining with the hypoxia-marker pimonidazole and that a correlation was observed for the expression of the Toustrup 15-gene hypoxia signature between the PDX tumours and donor clinical specimens [62,63]. However, two other studies have reported higher hypoxic fractions in PDX tumours compared to primary tumours based on pimonidazole immunostaining and a hypoxia score derived from ranking the median expression of genes in the Toustrup hypoxia gene signature [84,90]. These differences may be explained by the high inter-tumour variability in pimonidazole immunostaining [97,98,99] and different analysis methods for the Toustrup gene signature. Moreover, one of these studies did observe that the expression of endogenous hypoxia markers CA9, GLUT-1, and MCT-1 were similar in PDX and clinical tumours [90]. In other tumour types, higher levels of hypoxia have also been reported in PDX models that can be reversed by T_H_1 cell adoptive transfer [100]; but overall, the majority of studies report concordance in hypoxia between PDX and clinical tumours [91,101,102]. These data are promising given that subcutaneous cell line xenografts are typically more hypoxic than patient tumours, suggesting that PDX models may more accurately model clinical tumour hypoxia than cell line xenograft models [48,63].

The majority of HNSCC PDX models generated are HPV-negative rather than HPV-positive, as HPV-negative disease represents the greater clinical need, due to its greater frequency, worse prognosis, and reduced response to therapy relative to HPV-positive disease [8,23,24]. However, there is a clear need for clinically relevant models of HPV-positive HNSCC as there are few HPV-positive HNSCC cell line models available. While these HPV-positive HNSCC cell lines mostly recapitulate the genomic landscape of HNSCC tumours, they do lack some of the common genetic abnormalities present in clinical HNSCC tumours, particularly PIK3CA activating mutations [69,103]. A number of groups have developed HPV-positive HNSCC PDX models, which, with the exception of the 80% engraftment rate at the University of Wisconsin [81,85], generally have a lower engraftment rate than HPV-negative tumour models [80,82,83,84] (Table 1). Recent studies have revealed that these PDX models retain molecular markers and genomic features representative of HPV-positive clinical disease, including p16 staining, HPV-16 E6 and E7 RNA and DNA, and PIK3CA and NOTCH1 mutations [80,81,82,83,84,86]

Anecdotal reports of HNSCC PDX models predicting clinical response of the donor patient have been reported in four studies. Kang et al. report two PDX models, where the response to the pan-HER inhibitor afatinib mirrored the response of the donor patient, with one patient:PDX pair showing stable disease and the other partial response [79]. Both PDXs were also resistant to methotrexate, which both patients had previously been treated with. A third model was resistant to the pan PI3K inhibitor buparlisib as was the donor patient [79]. In a separate study, a PDX from a woman with heavily pre-treated HNSCC was treated with afatinib and metformin, with neither the PDX nor the patient’s tumour responding to therapy [93]. Campbell et al. describe a patient’s PDX with an EGFR amplification that responded to treatment with the MEK inhibitor trametinib initially followed by disease progression, consistent with the patient’s clinical outcome [92]. In the fourth study, a HNSCC patient developed recurrence following cisplatin-based chemoradiotherapy. A matched PDX model and cell line developed from the resistant tumour remained sensitive to gefitinib, so the patient was switched to gefitinib monotherapy and demonstrated remarkable regression within six weeks [88]. A second patient that progressed on cytotoxic chemotherapy was switched to erlotinib after the patient’s PDX showed a robust response, leading to tumour regression in the patient [88]. Another study compared response in PDX models to clinical responses in the donor patient, but PDX model treatment was restricted to monotherapy in contrast to combination with concurrent radiotherapy in the patient, so the clinical predictability of the models could not be accurately assessed [81]. Because of the small numbers involved and the likelihood of reporting bias, where similar studies that do not show associations between the patient and PDX response are not reported, the favourable clinical predictions in these studies need to be interpreted cautiously. However, there is evidence in larger cohorts of patients in other cancer types that PDXs can recapitulate the drug response of the donor patients [93,104,105] and can provide a useful platform for predicting population response in PDX controlled trials [106,107].

There are various limitations associated with the use of PDX models. A long time lag typically exists between initial engraftment and establishment of PDX tumours in large cohorts of mice for evaluation of drug sensitivity, which alongside the expense of developing these models, can limit their utility. The requirement for severely immunodeficient mice to allow for successful engraftment precludes a functional immune system in the mouse host. Interactions with the immune system influence tumour cell behaviour and provide a target for immunotherapies [108,109]. Co-engraftment of human haematopoietic stem cells and tumour specimens into immunodeficient mice (e.g., NSG, NSG-SGM3, MISTRG) can generate humanised PDX models, to enable interactions between PDX tumours and human immune cells, and thus incorporate the role of the immune system in tumour progression to more accurately recapitulate the human tumour microenvironment and allow for evaluation of immunotherapies [110]. Examples of humanised PDX models of HNSCC (XactMice) reflect this greater clinical relevance showing human immune cell infiltration in the tumour, altered cytokine and tumour gene expression, and increased lymphangiogenesis [111]. A further limitation of PDXs is their ability to model the considerable heterogeneity of the donor tumour. A small tumour fragment is unlikely to represent the spatial heterogeneity of the whole patient tumour and its implantation in mice may lead to the generation of a PDX subclone with different dynamics and response to therapy than the bulk of the primary tumour [112,113]. Further, the majority of HNSCC PDX tumours are engrafted subcutaneously on the flank of mice, which typically leads to a non-invasive phenotype contrary to the often highly aggressive and invasive behaviour of HNSCC tumours. Orthotopic implantation into the tongue, floor of mouth, or buccal mucosa can promote metastasis, but comes with animal welfare implications with regards to feeding and requirements for non-invasive imaging for tumour size measurements [114]. Finally, acquisition of copy number alterations (CNA) in subsequent generations of PDX tumours has led to questions about the reliability of PDXs to model the donor tumour [115,116]. However, an analysis of CNA in 1451 PDX models including HNSCC models, using five separate RNA- and DNA-based approaches to estimate copy number, rather than inferring CNA from gene expression microarray data, has contested these claims. Strong conservation of CNA from primary tumours through late-passage PDXs was observed with any differences in CNA comparable to spatial variation within patient tumours, and no systematic selection for CNA in cancer or treatment-related genes on engraftment or with passaging [117]. These findings do not argue against spontaneous tumour evolution [112,113], but rather suggest that the PDX mouse host does not drive systematic tumour evolution.

### 3.2. Patient-Derived 3D Cellular Models of HNSCC

Patient-derived 3D culture models offer an alternative approach to PDX models without the cost, time or animal ethics limitations of PDX models. Multicellular spheroid models of early passage HNSCC cell lines are common [118,119,120,121], but while these 3D models mimic oxygen and proliferative gradients of human tumours, they show limited histological fidelity to the donor tumour, like cell lines grown as monolayers, as a result of selection in culture [122]. In vitro expansion of cancer stem cells from dissociated primary HNSCC tumours grown in serum-free media leads to the development of tumour-derived spheroids [123,124,125]. These models are typically enriched for cancer stem cells as evidenced by high expression of stem cell markers and tumourigenicity in vivo [124,125], but the lack of serum precludes the survival of non-tumour cells that are an essential component of the human tumour microenvironment, while the success rate of tumour-derived spheroid formation can be low [125] (Table 2).

Advances in 3D culture technology have led to the development of tissue organoids, where 3D self-organising mini-organs can be grown in culture within a basement membrane gel from either pluripotent stem cells (PSCs) or adult stem cells (ASCs) [130,131,132]. This includes tumour cells isolated from tumour resections or biopsies from patients that grow with high efficiency in 3D culture as cancer organoids. Multiple organoid models for different types of cancer have been reported [133,134,135,136,137,138,139], including HNSCC at high success rates [126,128] (Table 2). Cancer organoids retain important structural and phenotypic properties of the cancerous tissue that they were derived from, offering useful benefits over conventional 2D cell cultures and 3D multicellular spheroids. These advantages, which have also been observed in HNSCC tumour organoids [126,127,128], include that they recapitulate more of the 3D organisation of the clinical tumour and that they can be grown long-term without genetic or functional changes [130,140]. Cancer organoids are suitable for modelling HPV-positive disease, either through culturing of an HPV-positive tumour specimen [128] or through HPV infection of organoids [126], and are suitable for genetic modification [141]. Cancer organoids can also be developed with high efficiency from patient tissue that has been grown in mice as PDX models and have similar morphology and drug sensitivity to the PDX models they were derived from [142]. As with PDXs, large biobanks of organoids are being established by various consortia (National Cancer Institute’s Patient-Derived Model’s Repository, Human Cancer Model’s Initiative) and contract research organisations (e.g., Hubrecht Organoid Technology, CrownBio, OcellO).

Cancer organoids offer promise in drug discovery as a link between 2D cell cultures and animal models for compound screening and identification of predictive biomarkers for precision medicine at a higher-throughput, with lower cost and shorter time-frames than PDX models [141]. Across multiple tumour types, tumour organoids have been shown to respond to most therapies in a largely consistent manner to the patients they were derived from [136,143,144,145,146,147], although, as with anecdotal reports in PDX models, reporting bias remains a potential concern. HNSCC organoid models show heterogeneous sensitivities to standard of care therapies [126,128] as would be expected clinically, while in a cohort of seven patients, the response to radiotherapy in the organoids correlated with the clinical outcome in most scenarios [126]. Due to the small numbers involved in these studies, prospective clinical trials in large numbers of patients are still required to confirm the ability of HNSCC organoids to predict clinical response. One such validation study is underway in the Netherlands to compare organoid response to patient outcome for standard first-line therapies in approximately 80 HNSCC patients (ONCODE-P2018-0003).

As with all preclinical models, there are limitations associated with tumour organoids. Although they accurately model epithelial cells, they lack a mesenchymal and immune component. Co-culture of tumour organoid systems with mesenchymal and/or immune cells have been reported [129,148,149,150], but these models are not yet well-established and supplementing the full diversity of human immune cells remains challenging. Patient tumour specimens can be contaminated by non-tumour cells which, when the specimens are grown as organoids, can outcompete the tumour cells and limit the effectiveness of the organoids at modelling human cancer [151]. Organoids have been reported to recapitulate hypoxic gradients [152]; however, their use for evaluating hypoxia-targeting drugs is challenging, since addition of the drug to the culture media causes physical disturbance that alters oxygen gradients in the media and will change the hypoxia status of the organoids [153]. Finally, a hallmark of cancer cells is anchorage-independent growth, yet, unlike spheroids or in vivo tumour models, tumour organoids are anchored to a basement membrane matrix. It remains unclear what influence the extracellular matrix has on organoid culture and its utility as a model for precision medicine [154].

## 4. Perspective on the Utility of PDX and Organoid Models for Precision Medicine of HNSCC

The evidence outlined above demonstrates that both PDX and organoid models of HNSCC are able to recapitulate the heterogeneity of clinical HNSCC, showing histopathological, genetic, and proteomic fidelity across multiple studies [75,76,77,78,79,85]. These models appear to have enhanced clinical relevance relative to cell line xenograft models or cell line monolayer cultures, but questions remain as to how well suited they are for the evaluation of new drug candidates and identification of predictive biomarkers to support precision medicine in HNSCC.

As previously discussed, there is anecdotal evidence that both HNSCC PDX models and organoids can predict the clinical response of the patients they originated from [79,88,92,93,126], suggesting their potential in avatar approaches of precision medicine. In this approach, PDX tumours or organoids act as avatars of the patient’s tumour they were derived from and response to multiple therapies can be compared to identify the best treatment strategy for the patient when their disease progresses on standard of care therapy [104]. Although various successful case studies have been reported, including some of those outlined in Section 3.1 [88,93,104], the moderate engraftment efficiency and long time taken for PDX models to be developed constrains their ability to influence clinical decision-making in many instances and has limited the widespread use of this approach. More commonly, PDX tumours are used to model human cancer at a population level, often as part of co-clinical trials, by using multiple PDX tumours of the same cancer type with heterogeneity in gene expression and resistance mechanisms to identify the best therapies for patients of that tumour type [106,107,110]. For organoids, the high success rate and shorter time frame of establishment make them more appropriate for an avatar approach. However, although preliminary studies have demonstrated the ability of cancer organoids to predict clinical response [136,143,144,145,146,147], further validation is required in larger prospective cohorts of patient-organoid pairs, particularly in HNSCC, to confirm their suitability as patient avatars [155].

The current lack of stromal and immune components in the majority of HNSCC PDX and organoid models limits their widespread use in precision medicine. Immune checkpoint inhibitors are approved for first- and second-line therapy of HNSCC [20,21,22], and so inclusion of a human immune component in PDX and organoid models is essential for them to be suitable to evaluate novel immunotherapy agents as well as novel drugs in combination with immunotherapy. Currently, despite a scarcity of available models, murine syngeneic models are most commonly used for evaluating immunotherapy agents in HNSCC [62,156], as they contain a functional immune system, with the caveat that the tumour and immune cells are not human. Tumour hypoxia is prevalent and a negative prognostic factor for patient outcome in HNSCC [28,157], so further investigation of the ability of HNSCC PDX models and organoids to model hypoxia and evaluate hypoxia-targeting drugs [62,63] is required. Preclinical models that can accurately model human tumour hypoxia with predictive biomarkers to identify the most sensitive models are essential to allow hypoxia-targeting drugs to progress into clinical trials for precision medicine of hypoxic HNSCC tumours [158]. Finally, although HPV-positive HNSCC has been increasing in incidence [159], until recently, few preclinical models have been available. The development of HPV-positive PDX models is encouraging [82,84,85] and may assist with precision medicine of HPV-driven HNSCC, but HPV-positive HNSCC organoids are still required [126,128].

## 5. Conclusions

Precision medicine offers the potential in improving therapeutic success to patients with HNSCC. The development of precision approaches will require an understanding of how the tumour microenvironment influences treatment responses, the co-development of biomarkers to accompany any therapeutic agents used in these approaches, and research tools that recapitulate the complexity of human tumours. A variety of HNSCC preclinical models exist, including a large number of HNSCC cell lines, which as monolayer cultures are ideal for basic biology studies and for early evaluations of pharmacological activity. However, due to their 2D organisation and in vitro selection, these culture models often poorly model the human tumour microenvironment, lacking stromal and immune cells and physiologically relevant oxygen gradients. Newer patient-derived preclinical models including PDX and organoid models of HNSCC are better able to recapitulate the heterogeneity of clinical HNSCC, with evidence of histopathological, genetic, and proteomic fidelity across multiple studies, and anecdotal reports of accurate predictions of clinical response. Overcoming current limitations to the lack of immune and/or stromal components through humanised mice or organoid co-cultures and prospective validation of their ability to predict clinical response will further their promise and utility as clinically relevant models for precision medicine in HNSCC.

## Figures and Tables

**Table 1:** Engraftment rate of head and neck squamous cell carcinoma (HNSCC) patient-derived xenograft models.

Institution ^1^	HPV-Negative		HPV-Positive		Unknown HPV-Status			
	No of Models	Engraftment Efficiency	No of Models	Engraftment Efficiency	No of Models	Engraftment Efficiency	Mouse Strain	Reference
University of Pennsylvania/Wistar Institute	11	56%	9	24%			NSG	[80,82]
Charité University Medicine, Germany	50	45%	2	14%			NSG	[83]
University of Colorado	16	59%	5	45%			Nu/Nu	[76]
University of Auckland, New Zealand	10	59%	1	100%			NSG	[62,63]
Aarhus University Hospital, Denmark.	5	50%	7	29%			NSG	[84]
University of Texas MD Anderson Cancer Centre					5	17%	Nu/Nu	[75]
University of Wisconsin	19	79%	8	80%			NSG	[81,85]
Roswell Park Comprehensive Cancer Center	3		3		17	60%	C.B-17 scid	[86,87]
Free University Hospital, Netherlands					30	26%	Nu/Nu	[74]
JE-UK Institute for Cancer Research/Yonsei University, South Korea	12	28%	3	16%			NOG	[79]
University of Pittsburgh	54	82%	4	80%	3	60%	NSG	[77]
Princess Margaret Cancer Centre, Canada	161	66%					NSG	[78]
A*STAR, Singapore					24	58%	NSG	[88]
Stanford University					16	53%	Rag2/Il2rg KO	[89]
Radboud University Medical Centre Nijmegen					18	60%	Nu/Nu	[90,91]
Washington University, St Louis					63	43%	NSG	[92]
Champions Oncology					36	68%	NSG	[93]

^1^ Primary institution(s) of first-named author.

**Table 2:** Success rate of patient-derived 3D cellular models of HNSCC.

Institution ^1^	Model	# of Models	Success Rate ^2^	Reference
Hubrecht Institute, Netherlands	Organoids from tissue	31	~60%	[126,127]
MD Anderson Cancer Center	Organoids from tissue	13	30%	[128]
Stanford University	Organoids from tissue	3	75%	[129]
Konkuk University, Korea	Spheroids from patient tissue	3	6.4%	[125]

^1^ Primary institution of first author ^2^ Success rate indicates the percentage of patient specimens that could be successfully established as 3D cellular models.
